# Further Evaluation of 1,25(OH)_2_D_3_-Glycosides Inclusion Timing for Reducing Clinical Bacterial Chondronecrosis with Osteomyelitis-Induced Lameness Incidence in Broilers

**DOI:** 10.3390/ani16162586

**Published:** 2026-08-19

**Authors:** Anh Dang Trieu Do, Ruvindu Perera, Woro Wulandari Kalanjati, Layla Al-Mitib, Zoe Zeman, Mian Rohaan Ahmed, Shashitej Mummadi, Gastón Castaño, Jenny Agudelo, Kathrin Bühler, Jan Dirk Van der Klis, Michael T. Kidd, Adnan Alrubaye

**Affiliations:** 1 Cell and Molecular Biology Program, University of Arkansas, Fayetteville, AR 72701, USA; rperera@uark.edu; 2 Department of Poultry Science, University of Arkansas, Fayetteville, AR 72701, USA; worok@uark.edu (W.W.K.); mkidd@uark.edu (M.T.K.); 3 Directorate of Risk Management, Animal Quarantine, Indonesian Quarantine Authority, Jakarta 12550, Indonesia; 4 Department of Biological Sciences, University of Arkansas, Fayetteville, AR 72701, USA; lhalmiti@uark.edu (L.A.-M.); ma143@uark.edu (M.R.A.); mummadi@uark.edu (S.M.); 5 Department of Animal Science, University of Arkansas, Fayetteville, AR 72701, USA; zzeman@uark.edu; 6 Animal Science Faculty, Corporación Universitaria de Santa Rosa de Cabal (UNISARC), Santa Rosa de Cabal 661020, Risaralda, Colombia; gcastano@uark.edu (G.C.); jagudelo@uark.edu (J.A.); 7 Herbonis Animal Health GmbH (Innovad Group), 4302 Augst BL, Switzerland; k.buehler@innovadgroup.com (K.B.); jd.vanderklis@innovadgroup.com (J.D.V.d.K.)

**Keywords:** poultry, leg, bone, vitamin D, infection, additive

## Abstract

This experiment compared 14-day and 28-day inclusion schedules of 1,25(OH)_2_D_3_-glycosides in the broiler feed on the reduction of experimental clinical bacterial chondronecrosis with osteomyelitis (BCO) lameness, using the aerosol transmission induction model. Eight hundred and forty Cobb 500 chicks were randomly allocated to each of the following treatments at 60 chicks/pen as the initial stocking density and reared over 56 d: T1 (Positive Control-PC, 2 wire-flooring pens)—Basal diet (7166 IU/kg Vit-D3); T2 (Negative Control-NC, 4 litter pens)—Basal; T3 (4 litter pens)—Basal + 14d 1,25(OH)_2_D_3_-glycosides (1 µg/kg feed); and T4 (4 litter pens)—Basal + 28d 1,25(OH)_2_D_3_-glycosides (1 µg/kg feed). There were three feeding phases: Starter (d0–21), Grower (d22–42), and Finisher (d43–56). Clinical lameness assessment was conducted daily from d21, followed by euthanasia and necropsy of clinically lame birds to determine BCO lesion severity. Cumulative lameness incidence rates between treatments were assessed using binomial logistic regression analysis in a generalized linear mixed model. All statistical significance was determined at *p* < 0.05. Cumulatively, T4 (55.5%) was significantly lower in clinical lameness incidence compared to T2 (85.0%, relative decrease of 34.71%; *p* < 0.0001), while T3 (85.5%) was not significantly different (*p* = 0.89). These results further affirm efficacy of the 28-day inclusion period and the rate of 1,25(OH)_2_D_3_-glycosides against experimental clinical BCO lameness incidence.

## Simple Summary

Bacterial lameness in broiler chickens remains a significant issue affecting global poultry production economics and animal productivity. Experimentally, the early inclusion of supplemental vitamin D_3_ glycosides in the first four weeks of broiler life has been documented as an effective preventive feeding strategy against this disease. This study further evaluated the efficacy of this established inclusion timing compared to a shorter period of 14 days in terms of clinical lameness outcomes. Results at the end of the experiment further affirm the efficacy of a 28-day inclusion period of supplemental vitamin D_3_ glycosides compared to known evaluated schedules.

## 1. Introduction

Bacterial chondronecrosis with osteomyelitis (BCO), along with its associated lameness, remains a major challenge facing the international poultry industry [1,2,3]. The disease stems from the disproportionate weight accumulation and muscling rate of the modern fast-growing broiler compared to the slow development rate of its skeletal system—particularly of the leg bones (tibia, femur), which have remained relatively stable for the past 20 years despite advancements in genomics and genetic selection for improved leg health [4,5,6]. The heavy body weight exerts tremendous stress on the immature leg bones, especially from the third week of life onward when daily weight gain becomes markedly pronounced [2]. This leads to lesion-forming microfractures and osteochondritic clefts within the proximal femoral physis, proximal tibial epiphysis, and tibial physis. Consequently, translocation of pathogenic bacteria in the bloodstream—whether acquired through respiration, ingestion, or from the animal’s internal environment—results in deposition and focal infection at these lesions, which worsens as necrosis takes place, leading to lameness and eventual death of the bird if not culled. Considering modern broiler production practices with high stocking density rates, transmission risks of BCO are heightened from increased environmental and pathogenic exposure, as well as other predisposing factors, such as stress. In fact, industry communications have reported infection rates of up to 10–15% during heavy outbreaks [7,8]. At the same time, focal delivery of treatments in the medullary system to damaged bone tissue is inherently difficult [9]. Coupled with the short-lived production cycle of the modern broiler (averaging 42 days per standard flock), BCO is therefore both intractable and economically infeasible to treat on an individual basis, leaving culling losses as the only practical solution to prevent its spread. As such, preventative measures for BCO have gained increased attention in both production and research settings due to its emerging global prevalence and significant economic impact on the poultry industry at large [10,11,12,13].

Feed additive usage intended to improve broiler productivity and health has been a popular choice among producers, the beneficial effects of which are well-documented in current literature, ranging from increases in general performance parameters to conferring humoral, antiviral, and antibacterial immunity against diseases [14,15,16,17,18]. In recent years, 1,25(OH)_2_D_3_ has emerged as a promising candidate additive in BCO preventative research. This biologically active form of vitamin D_3_ is known in the literature as a key player in bone homeostasis via calcium and phosphorus absorption regulation [19], as well as host immunological functions [20]. As a metabolite supporting bone health, its supplemental inclusion has been shown to decrease broiler tibial dyschondroplasia incidence and severity [21,22], in addition to increased egg production and eggshell quality [23]. In terms of immunomodulation, supplemental 1,25(OH)_2_D_3_ supports increased in vitro innate immune response to immunosuppressive pathogens such as infectious bursal disease virus [24], induces heightened expression of avian β-defensins in embryonic epithelial cells [25], and increases performance and immune functions at the third and sixth week of age [26]. With regard to BCO, experimental supplementation of 1,25(OH)_2_D_3_-glycosides at an inclusion rate of 1 µg 1,25(OH)_2_D_3_-equivalent/kg of feed for the first four weeks of broiler life has shown encouraging results, with a reduction of 53.7% in clinical lameness cases [27], providing an effective and economical solution to preventive measures against BCO as a disease.

Despite the beneficial effects of early 1,25(OH)_2_D_3_ inclusion in the broiler diet, as well as its optimal concentration, there still remains a significant gap in the literature regarding its inclusion timing against BCO or other notable diseases, potentially limiting its application in the poultry industry. For instance, a comprehensive efficacy profile may be invaluable to producers seeking to optimize cost–benefit approaches in their operations, thus encouraging its widespread adoption as an effective feed additive. Leveraging the significant documented protection provided by supplemental 1,25(OH)_2_D_3_-glycosides against clinical BCO lameness, this study thus aimed to further investigate whether a shorter inclusion period (two weeks compared to four) can confer similar protective effects in experimental broilers. The study was conducted using the aerosol transmission model of BCO induction [28] with the working hypothesis that a four-week inclusion period will confer a similar reduction in clinical lameness as previously documented, and shortening this period will reduce this effect. The insights obtained may be of significant interest to the poultry industry in their application toward effective mitigation strategies against BCO-associated clinical lameness, further contributing to efforts toward sustainable global poultry production.

## 2. Materials and Methods

### 2.1. Institutional Animal Care and Use Committee

The study was approved and conducted under the University of Arkansas Agricultural Institutional Animal Care and Use Committee (Ag-IACUC) protocol #25052 on 12 August 2025.

### 2.2. Environment, BCO Induction Model, and Treatment Allocation

#### 2.2.1. Housing Environment

The study was conducted at the University of Arkansas Poultry Research Facility Complex (House 365E) in Fall 2025 and lasted 56 days. On d1 of the study, 840 Cobb 500 male broiler chicks from a breeder line were procured from a local hatchery and randomly allocated to fourteen 1.52 m × 3.04 m pens, including two raised wire-flooring pens (1.3 × 2.54 cm mesh, 0.063 gauge) [29] and twelve litter-flooring pens, at a density of 60 chicks per pen (0.077 m^2^/bird). On d14 of the experiment, the number of birds per pen in all treatments was uniformly reduced to 50 to maintain a stocking density of 0.092 m^2^/bird per IACUC specifications. Culled birds per pen on d14 (apart from previous culls and mortalities, if any) were selected altogether based on general clinical observation deviating from the norm (lethargy, weakness, morbidity, injuries, and/or stunted growth), followed by random selection until a final number of 50 clinically normal birds/pen was reached, but were otherwise not specifically noted categorically. Research personnel performing bird selection were blinded to treatment details to reduce potential bias.

#### 2.2.2. Aerosol Transmission Model of BCO Induction

The aerosol transmission of BCO induction has been previously published [28]. Briefly, an “infection seeder” group (Positive Control-PC) consisting of two (2) wire-floor pens is situated at one end of the housing environment, upstream of ventilation airflow, with an empty “buffer zone” separating the group from the remaining groups. The main treatment groups of interest (including the Negative Control-NC) consist of broilers reared on normal litter flooring and line the remaining length of the house, directly downstream of the same ventilation airflow. Mechanistically, shed and aerosolized etiological agents from the PC group, which faces significant physiological stress and rapidly develops clinical BCO lameness, can be transmitted by the aforementioned ventilation airflow to the remaining treatment groups on litter flooring. This model of BCO induction inherently does not rely on additional bacterial challenge administration but rather exploits the dissemination of etiological agents naturally acquired (and exacerbated) by the seeder population in a similar fashion to horizontal transmission in an industrial setting [28].

The light schedule was set at 23L:1D for the entire duration of the experiment. House settings were automatically regulated to reach bird thermoneutral temperatures following a standard brooding schedule: 32 °C for days 1–3, 31 °C for days 4–6, 29 °C for days 7–10, 27 °C for days 11–14, 24 °C for days 15–17, and 21 °C for the remaining duration of the 56-day experiment. Birds had ad libitum access to feed and water for the duration of the study.

#### 2.2.3. Experimental Treatment Allocation

There were three (3) feeding phases in all treatments: Starter (d1–d21, crumbles), Grower (d22–d42, pellets), and Finisher (d43–d56, pellets). These basal diets contained 7166 IU vitamin D_3_/kg of feed provided by the vitamin premix. 1,25(OH)_2_D_3_ was supplemented as glycoside by adding Panbonis^®^ (Herbonis Animal Health GmbH, Augst BL, Switzerland) to the control diets. Panbonis^®^ contains the dried and ground leaves of the plant *Solanum glaucophyllum* and provides 1 µg 1,25(OH)_2_D_3_ in glycosidic form per kg of feed at an inclusion rate of 100 g/metric ton (MT) of feed as per manufacturer specifications. Birds were assigned to four treatment groups: Treatment 1 (Positive Control-PC): two wire-floor pens receiving basal diet for all 56 days; Treatment 2 (Negative Control-NC): four litter-floor pens receiving basal diet for all 56 days; Treatment 3: four litter-floor pens receiving basal diet supplemented with 100 g of Panbonis^®^/MT feed (0.01%) from d1–14 and basal for the remainder (d15–56); and Treatment 4: four litter-floor pens receiving basal diet supplemented with 100 g of Panbonis^®^/MT feed (0.01%) from d1–28 and basal for the remainder (d29–56).

In treatments with Panbonis^®^ inclusion, the additive was added in place of a filler ingredient consisting of inert sand. As Panbonis^®^ inclusion only lasted from d1 to d14 (partial Starter phase) for T3, and d1–d28 (entire Starter phase and partial Grower phase) for T4, this phase was further split into two mini-phases to facilitate normalized nutrient levels across treatments in the Grower phase: Grower 1 (d22–d28) with filler space, and Grower 2 (d29–42) without.

A summary of treatment allocation for all experimental diets is provided in Table 1. The composition of the basal diet in all phases of the study is detailed in Table 2.

**Figure 1:**
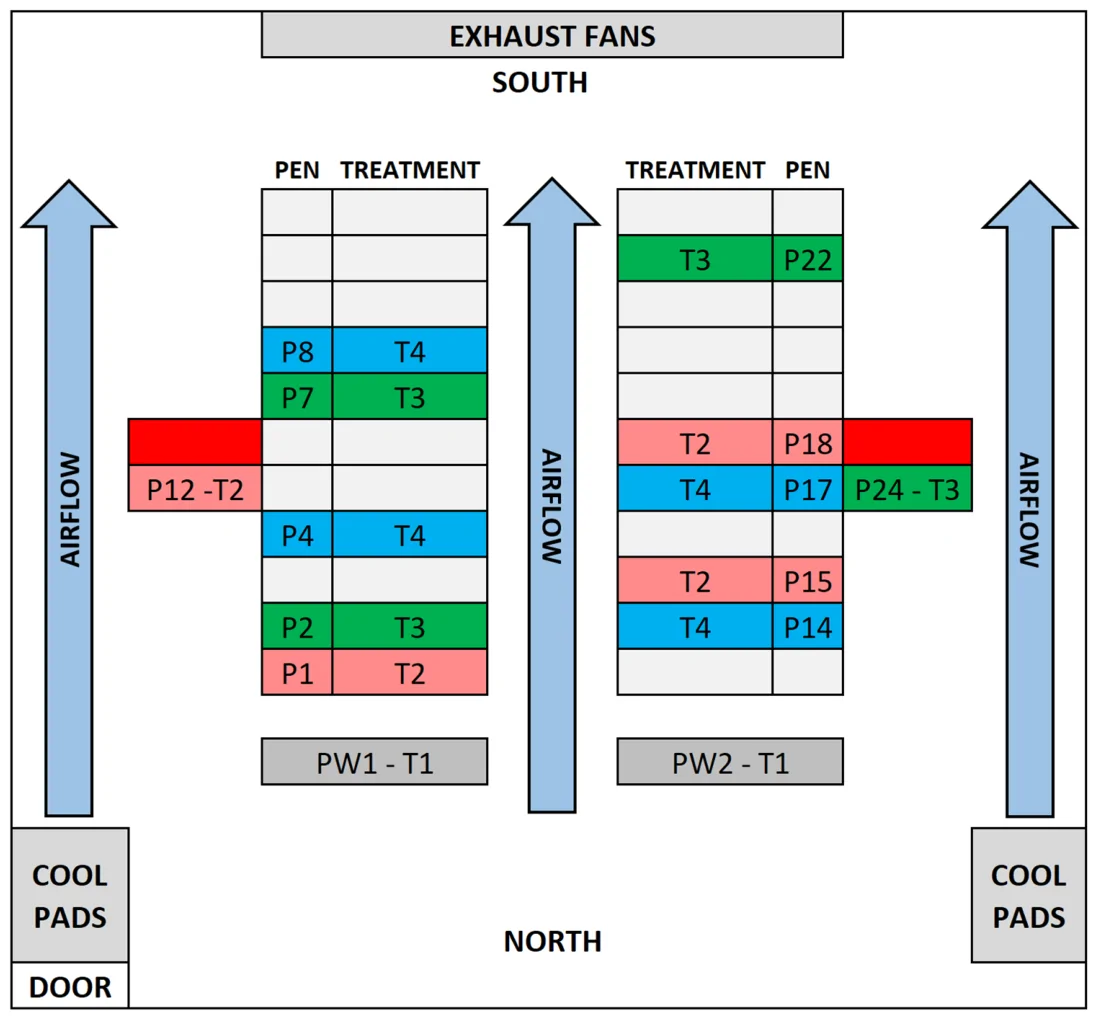
Barn layout of the study, with color-coded treatment pen allocation and descriptive elements involved in the aerosol BCO induction model (not to scale). Unmarked red cells indicate unused pens. Unmarked light grey cells indicate pens occupied by other treatment population(s) not related to the study.

Figure 1 visualizes the barn layout of the study. Treatment pens were randomly allocated throughout the barn’s length, with no pens of the same treatment placed adjacent to or across from each other. Facilitated ventilation airflow was confirmed to reach all treatment pens before the start of the study.

### 2.3. Clinical Lameness Evaluation

**Figure 2:**
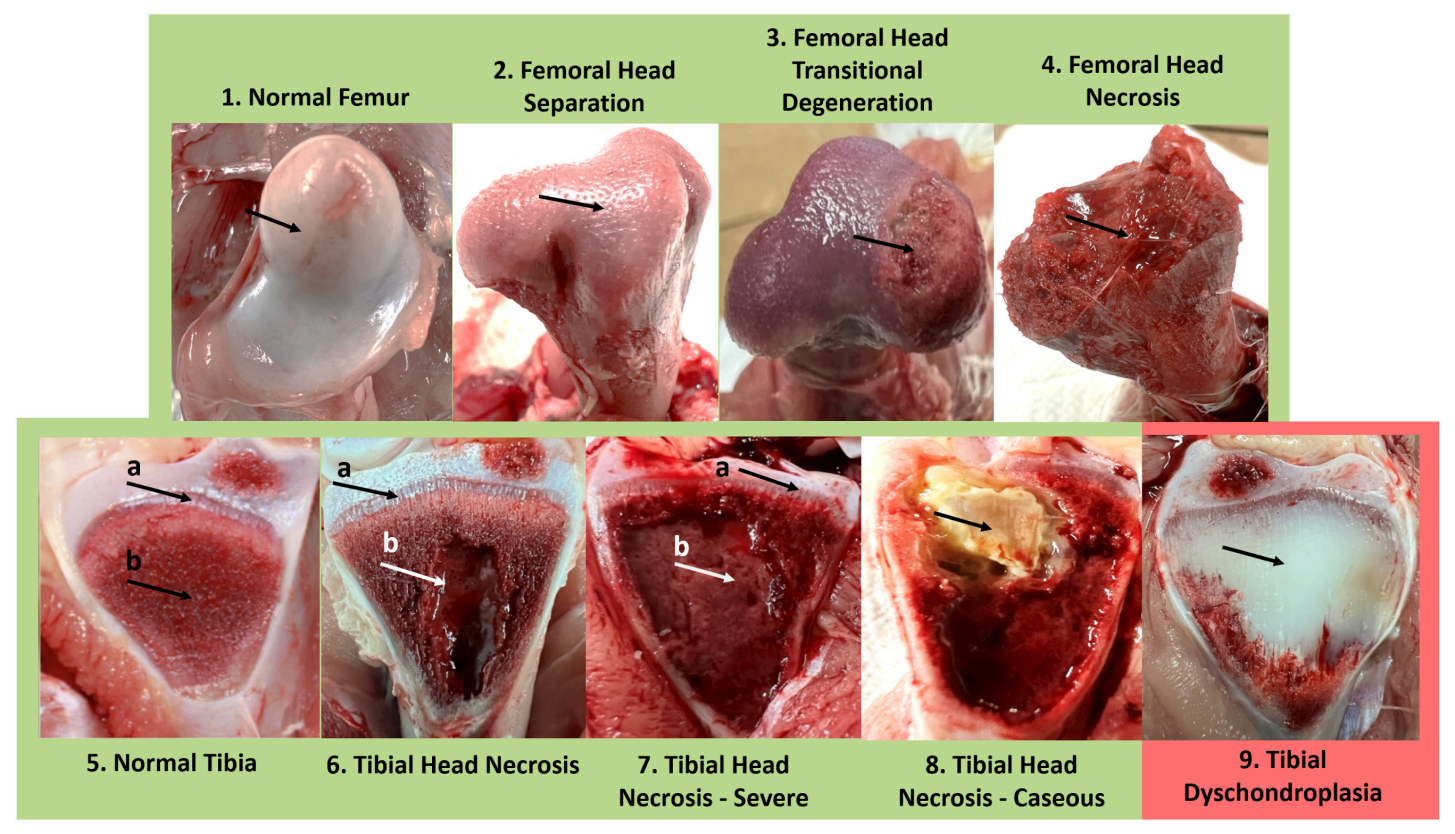
Adapted with permission from Do et al. [31]. Examples of typical femoral (1–4) and tibial (5–9) lesion categories observed during gross lesion evaluation. Lesions shaded in green are considered part of BCO severity progression, while those shaded in red are not. Arrows provided to indicate hallmark characteristics: 1. Normal proximal femoral head state with intact epiphyseal articular cartilage; 2. Proximal femoral epiphysis surface separated from cartilage that remains in the acetabulum; 3. Separated proximal femoral epiphysis with varying degrees of damage (moderate lesion here with fibrinonecrotic exudate); 4. Extreme damage to fracture of weakened proximal femoral epiphysis and physis upon disarticulation of the femur; 5. Normal state of proximal tibia with clearly defined physeal growth plate (a) and firm cancellous bone (b); 6. Necrotic state of the proximal tibia, still with clearly defined physeal growth plate (a) but damaged cancellous bone, replaced with a necrotic void of various sizes (b); 7. Severe necrotic state of the proximal tibia, with the physeal growth plate (a) encroached upon by large necrotic void (b); 8. Necrotic state of the proximal tibia with additional caseous exudate, marking extensive bacterial infiltration region; 9. Proximal tibial head afflicted with tibial dyschondroplasia, marking abnormally large region of cartilage instead of cancellous bone but is otherwise unrelated to BCO pathology and is not considered a BCO lesion. Presence of at least one pathognomonic lesion (excluding 1, 5, and 9) in a necropsied bird indicates BCO pathology.

From d21 of the experiment onward, clinical lameness in each treatment pen was evaluated by gently encouraging birds to move brief distances by trained research personnel. Birds exhibiting difficulty in movement, limping gait, or otherwise completely unable to move were diagnosed as clinically lame. These birds were removed from each pen and humanely euthanized in an air-tight container with a gas inlet, which was then filled with approximately 35–50% CO_2_ gas by volume per standards allowed by the American Veterinary Medical Association (AVMA) [30]. All birds euthanized via rapid CO_2_ inhalation were confirmed dead following a brief waiting period (1 min) by research personnel. Additionally, while treatments were labeled per pen to ensure accurate recordkeeping, treatment details were not disclosed to trained research personnel evaluating clinical lameness diagnosis. Post-mortem birds were necropsied to assess and confirm presence of pathognomonic BCO lesions of the proximal femoral and tibial heads, as per Wideman [2]. Typical femoral and tibial lesions observed at this stage are visualized in Figure 2, following the severity scale as per Do et al. [31]. Except for one instance, all remaining cases of diagnosed clinical lameness in the study were confirmed as BCO-induced with at least one gross subclinical lesion pathognomonic of BCO pathology.

In addition to lameness cases, upon daily bird health check starting from d1 of the study, all cases of mortality, as well as sampling and health/welfare euthanasia, were removed from their respective pens following the evaluation categories outlined in Table 3. In the latter, birds were euthanized in the same manner as stated above (CO_2_ inhalation).

All cases of clinical lameness, culls, and mortality were recorded throughout the entirety of the experiment. However, only cases within the evaluation period (d21–d56) are considered for clinical lameness analysis. There were no mortality or cull cases between d14–d21. At the end of the trial on d56, all remaining non-lame birds were recorded as clinically healthy.

### 2.4. Statistical Analysis

While presented in some statistical tests for context, T1 was excluded from result interpretation due to significant differences in pen design, conditions, and purpose, with any presented statistics provided only for contextual and descriptive purposes. Statistical analyses were conducted using R v4.6.1 (R Foundation for Statistical Computing, Vienna, Austria).

For cumulative lameness analysis, binomial logistic regression of cumulative lameness incidence between treatments was conducted using a generalized linear mixed model (GLMM) in package glmmTMB [32] and further processed with packages modelbased [33] and gtsummary [34], with presence of clinical lameness (Lame = YES) as the measured outcome. Sampling, health/welfare culls, and mortalities were not considered clinical lameness in diagnosis, as clinical lameness was either: (a) not observed during sampling selection, (b) superseded by other clinical signs, or (c) indeterminable due to death. Thus, all birds that were not explicitly diagnosed as clinically lame during the study’s evaluation period were not considered lame (Lame = NO). Diet treatments are considered the fixed effect, clustered by pens. The reference level for statistical analyses is T2 (NC).

Additionally, a time-to-event (survival) analysis of BCO-associated clinical lameness between T2, T3, and T4 from d21 to d56 was conducted. Right-censoring was applied to all birds that were not defined as clinically lame (Lame = NO) due to the same reasons listed, as it was not possible to evaluate clinical lameness development in these individuals past their removal dates. For birds that were removed from the population (sampling, health/welfare culls, mortalities) before the end of the evaluation period on d56, censoring was applied on day of removal. All remaining clinically healthy birds at the end of the experiment were also censored on the last day of the evaluation period (d56). The censored dataset is included in Table S1. Visualization of Kaplan–Meier curves and pairwise log-rank statistical significance testing were done using R packages survival [35] and survminer [36]. Further Cox proportional hazards regression modeling with pen clustering was conducted using package autoReg [37]. All statistical significance was defined at *p* < 0.05.

## 3. Results

**Figure 3:**
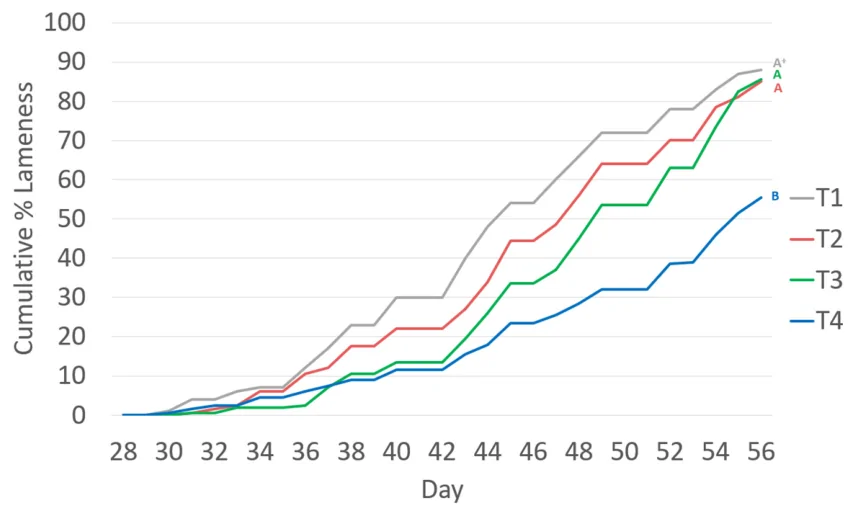
Cumulative lameness incidence of all treatments in the study. Note: Non-connecting superscripts denote significant difference. ^+^ T1 statistics provided for contextual purposes only. Treatments: T1—Positive Control (basal diet); T2—Negative Control (basal diet); T3—Basal + 1 µg/kg feed 1,25(OH)_2_D_3_-glycosides d1–d14; T4—Basal + 1 µg/kg feed 1,25(OH)_2_D_3_-glycosides d1–d28.

The cumulative incidence of lameness per treatment from d28 to d56 of the experiment is presented in Figure 3 and tabulated in Table 4. Raw counts of clinically lame, mortality/cull, and healthy (survivor) cases are tabulated in Table 5 and summarized in percentages in Table 6. The first cases of clinical lameness were observed on d30 in T1 and T4.

**Figure 4:**
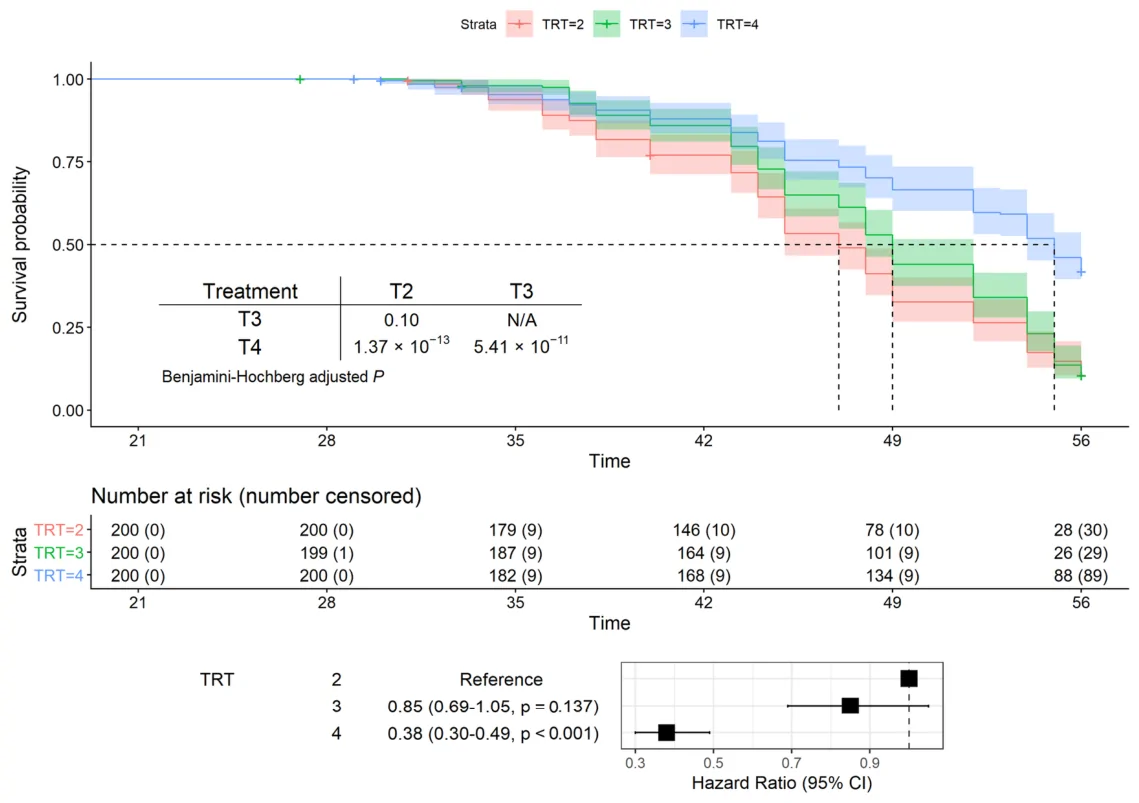
Time-to-event (survival) analysis of clinical lameness incidence between T2, T3, T4 from d21 to d56. T1 excluded from analysis. Top: Kaplan–Meier survival curves; Bottom: Cox hazard ratio plot with pen clustering. Each step down in Kaplan–Meier curves signifies clinical lameness events. Plus (+) symbols signify right-censored events. Treatments: TRT2—Negative Control (basal diet); TRT3—Basal + 1 µg/kg feed 1,25(OH)_2_D_3_-glycosides d1–d14; TRT4—Basal + 1 µg/kg feed 1,25(OH)_2_D_3_-glycosides d1–d28.

As expected, by the end of the study on d56, T1 (PC), T2 (NC), and T3 had reached high cumulative lameness incidence rates at 88.0%, 85.0%, and 85.5%, respectively. The lowest cumulative incidence rate was observed at 55.5% in T4. Figure 4 visualizes the time-to-event (survival) analysis of BCO-induced clinical lameness incidence between T2, T3, and T4 in the evaluation period from d21–d56 in both Kaplan–Meier curves and Cox proportional hazards regression. T4 was significantly different from T2 and T3 (Kaplan–Meier log-rank pairwise Benjamini–Hochberg adjusted *p* = 1.37 × 10^−13^ and 5.41 × 10^−11^, respectively). With T2 as the reference level, T3 has a similar hazard ratio of 0.85 (*p* = 0.137), while T4 was significantly lower at 0.38 (*p* < 0.001).

Comparative binomial logistic analysis of total cumulative lameness incidence between treatments at the end of the experiment, with T2 (NC) as the reference level, is tabulated in Table 7. Excluding T1, T4 was significantly lower in absolute cumulative lameness incidence rate compared to T2 by 29.5% (relative decrease of 34.71%; *p* < 0.0001), while T3 was not significantly different from T2 (0.5% difference; *p* = 0.89).

**Figure 5:**
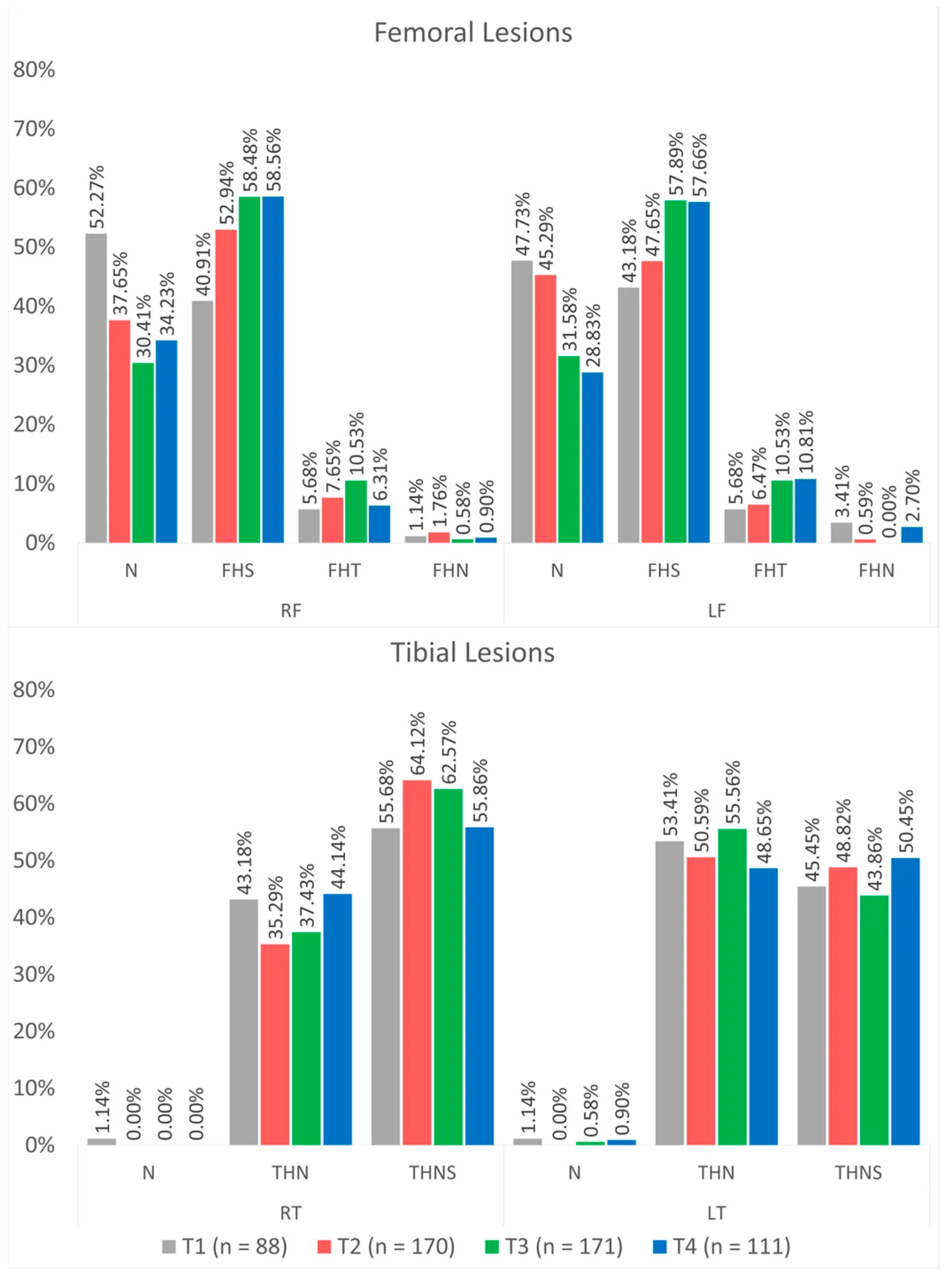
Femoral and tibial lesion ratings for birds diagnosed as clinically lame. The percentage of lame birds with the indicated BCO lesion(s) is plotted for each of the bones by treatment. Note: *n* = number of clinically lame birds per treatment; RT—Right Tibia, LT—Left Tibia, RF—Right Femur, LF—Left Femur, N = Normal, FHS = Femoral Head Separation (epiphyseolysis), FHT = Femoral Head Transitional degeneration, FHN = Femoral Head Necrosis, THN = Tibial Head Necrosis, THNS = Tibial Head Necrosis Severe. THNC (Tibial Head Necrosis Caseous) excluded due to absence of cases. Tibial dyschondroplasia (TD) excluded (not BCO lesions). Treatments: T1—Positive Control (basal diet); T2—Negative Control (basal diet); T3—Basal + 1 µg/kg feed 1,25(OH)_2_D_3_-glycosides d1–d14; T4—Basal + 1 µg/kg feed 1,25(OH)_2_D_3_-glycosides d1–d28.

The raw cumulative counts of femoral and tibial BCO lesions are tabulated in Table 8, with visualization presented in Figure 5.

No apparent trend was found among all treatments in terms of cumulative lesion severity distribution. For femoral lesions, the highest prevalence was femoral head separation (FHS) in both left/right femur and was shared between T3 and T4 (58.48%, 58.56%, 57.89, and 57.66%, respectively). For tibial lesions, the highest incidence was severe tibial head necrosis (THNS) in T2 at 64.12%. No case of severe tibial head necrosis with caseous exudate (THNC) was observed in the study.

## 4. Discussion

As mentioned, while some statistical analyses and analyses of T1 were presented for context, discussion of results from this treatment was excluded due to the different pen design, conditions, and replicate allocation. As reported in Table 4 and Figure 3, the first observable clinical lameness cases began on d30 of the experiment. While there were no clear visual trends within the d21–d38 period, T2 and T3 had similar trajectories from this point onward until the end of the experiment, while T4 markedly accumulated clinical lameness cases more gradually. The duration of 1,25(OH)_2_D_3_-glycosides supplementation had a significant effect on the cumulative BCO-induced clinical lameness incidence. Compared to the NC group, inclusion for the first two weeks of the experimental broiler’s life had no distinguishable difference in cumulative lameness outcomes (T2: 85.0% versus T3: 85.5%; *p* = 0.89). In contrast, continuous supplementation for an additional two weeks resulted in significantly lower cumulative lameness incidence that continued until the end of the experiment at d56 (T2: 85.0% versus T4: 55.5%, relative reduction of 34.71%; *p* < 0.0001). Time-to-event analysis of clinical BCO lameness (Figure 4) further corroborates these findings, with a significant difference between T4 and both T2 and T3 (Kaplan–Meier *p* < 0.0001; Cox hazard ratio *p* < 0.001), but not the latter two (*p* > 0.05). The median survival times for T2, T3, and T4 are d47, d49, and d55, respectively. Post hoc marginal contrast analysis also showed that T3 and T4 are significantly different (*p* < 0.001).

These results are in line with previous findings, where supplementation of 1,25(OH)_2_D_3_-glycosides in the broiler diet for the first four weeks of life resulted in similar decreased BCO-associated clinical lameness incidence as a full eight-week inclusion period under the same experimental conditions [27]. We hypothesize this inclusion period provided sufficient support to help the broiler cope with stressful developmental changes, which a two-week period may have failed to achieve. The modern broiler experiences the most pronounced body weight gain during the third to fifth week of life, a period that may be connected to weight-related BCO pathogenesis mechanisms [2,3]. Coupled with physiological and environmental stress, in addition to leg bone development incommensurate with body weight gain in the same period [4,38], there are grounds to believe that this period of the broiler’s life is crucial to effective control of BCO development. For this reason, inclusion periods shorter than four weeks may not be sufficient to confer protection against BCO pathogenesis and subsequent disease development. Future research is thus warranted to examine the additive’s mode of action with regard to inclusion timing, such as effect persistence post-withdrawal, in greater detail. Additionally, we hypothesize that effective inclusion of 1,25(OH)_2_D_3_-glycosides may reduce clinical lameness incidence associated with BCO via reduction of causative bacteremia through improved epithelial integrity [39,40] and/or synthesis of antibacterial peptides [41,42], which provides a mitigative or preventive effect. As the active and functional form of vitamin D3, inclusion of 1,25(OH)_2_D_3_-glycosides may also implicate improved immune functions and early bone development, both keystone aspects in postulated BCO pathogenesis [2], and may offer alternative insights into the observed reduction of clinical lameness incidence.

Insofar as BCO lesion severity is discussed, no clear differences between treatments in lesion distribution of clinically lame birds were observed in this experiment (Figure 5), similar to other reports in BCO pathology and preventive research [27,43]. However, this evaluation remains relatively nebulous and should be approached with caution. From the current literature, lesions may begin development unilaterally and irrespective of age well before clinical signs [2,3]. Compounded with the currently limited scope for rapid and accurate temporal assessment of lesion development in live birds, this further adds to the difficulty in evaluation of treatment impact on lesion development. Much closer examination and methodological development are thus required to elucidate accurate treatment impact on BCO lesions.

While these results further affirm the efficacy of a four-week inclusion of 1,25(OH)_2_D_3_-glycosides among evaluated inclusion schedules, some challenging aspects related to sampling protocols and their impact on analyses pertaining to population dynamics should be evaluated in future studies. In this study, nine birds from T1 and eight birds from each of T2–T4 were sacrificed on d29 and d33, reflecting additional sampling protocols. Cumulatively, sacrificed birds constituted 9% of T1’s population (*n* = 100) and 4% in each of T2–T4 (*n* = 200/treatment). While this did not have a significant impact on treatment populations (particularly in T2–T4), careful consideration must nonetheless be given to constraints regarding additional sampling, as population analyses to determine endpoint cumulative lameness outcomes—such as those in this current study—are sensitive to the loss of animals sacrificed for tissue collection, since each bird has an independent chance to develop clinical lameness. While a low replicate count may not reflect accurate representation of treatment effects, too many sampled animals may also be detrimental, and future investigators should strive to reach a delicate balance in the number of biologically relevant replicates—for example, by reducing the number of sample collection timepoints or using non-terminal tissue. In addition to sampling considerations, the proper induction model must also be assessed according to research goals. Moreover, even though the aerosol transmission model is highly translatable thanks to its capacity for horizontal transmission of BCO infection without direct human intervention, its mechanisms are inherently tied to running ventilation and are thus subject to seasonality [44,45]. Depending on the topic of investigation, researchers should carefully consider whether the use of such a model is suitable compared to other direct methods (such as oral gavage, injection, or feed/drinking water challenge). Finally, while there were no significant differences in average surviving broiler weights between the negative control and treatment populations on d56 (Table S2), it should be noted that this comparison is limited only to surviving birds, as the experiment was not designed with a priori performance parameters (such as feed intake and weight gain) in consideration. As such, no solid conclusions can be drawn regarding treatment effect on broiler performance in this experiment. Future research aiming to evaluate this aspect in tandem with clinical lameness evaluation, however, should exercise caution in experimental design to account for the rapid and high loss of birds in later stages of the study.

With these findings, several aspects remain that may guide future researchers investigating similar topics. To address and elucidate lesion formation and progression between varying supplement inclusion periods, future studies should emphasize noninvasive temporal development of the femur and tibia, such as through computed tomography and dual-energy X-ray absorptiometry scanning of live birds. In the same vein, assessment of the femur and tibia—such as geometrical measurements, mineral composition, density, and breaking strength—may provide a more complete picture in terms of bone health commonly associated with circulating levels of 1,25(OH)_2_D_3_. As 1,25(OH)_2_D_3_ is an important active metabolite contributing to host immune function [46], further investigation of temporal immune responses is also of high importance to determine the glycoside’s mode of action—particularly in an environment that perpetually exposes experimental animals to aerogenous bacterial spread [28]. Finally, the currently accepted model of BCO pathogenesis intimately involves loss of function in tight junction (TJ) proteins responsible for gastrointestinal and respiratory epithelial integrity [2,39,40], leading to subsequent translocation events of pathogenic bacteria to colonization sites. Measurements of mRNA expression levels in TJ genes like occludin (*OCLN*) or the claudin (*CLDN*) family, in addition to epithelial integrity assays (such as the fluorescein isothiocyanate dextran assay; FITC-d) from intestinal and respiratory tissues, can further augment the understanding of BCO pathology for effective development of preventive strategies.

## 5. Conclusions

Leveraging previously documented positive effects in the early four-week supplementation of 1,25(OH)_2_D_3_-glycosides at an inclusion rate of 1 µg/kg feed against clinical BCO lameness outcomes, this study investigated whether an even shorter inclusion period of the same dose in the experimental broiler diet would confer similar benefits, using the aerosol transmission of BCO induction. Binomial regression analysis of cumulative BCO lameness at the end of the study showed that the four-week period was effective in reducing clinical lameness outcomes consistent with previous studies, while the two-week period was ineffective in this goal. Future research should focus on parameters measuring bone health, lesion formation, and immunological responses, as well as respiratory and intestinal epithelial integrity, to elucidate a complete picture of BCO pathology upon which effective management and preventive strategies can be developed.

## Figures and Tables

**Table 1:** Experimental treatments and treatment allocation.

Treatment	Pens	Description	Diet	Flooring	*n*
T1-PC	2	Positive Control	Basal (d1–d56)	Wire	100
T2-NC	4	Negative Control	Basal (d1–d56)	Litter	200
T3	4	14d Inclusion	Basal + 1 µg/kg feed 1,25(OH)_2_D_3_-glycosides (d1–d14); Basal (d15–d56)	Litter	200
T4	4	28d Inclusion	Basal + 1 µg/kg feed 1,25(OH)_2_D_3_-glycosides (d1–d28); Basal (d29–d56)	Litter	200

Note: *n* = Number of animals per treatment post-reduction on d14. In total, 19 birds from T1, 31 birds from T2, 29 birds from T3, and 32 birds from T4 were removed on d14, excluding previous culls and mortalities from d1–d13.

**Table 2:** Composition of the basal diet used for the study.

Ingredients	Starter (d1–d21) (%)	Grower 1 (d22–d28) (%)	Grower 2 (d29–d42) (%)	Finisher (d43–d56) (%)
Corn-7.3% CP	56.74	62.70	63.39	67.62
Soybean meal-47.5% CP	32.90	28.47	28.40	25.58
Pork meat & bone meal	5.00	3.50	3.50	-
Oil, Poultry fat	2.91	3.07	2.81	3.90
Dicalcium phosphate	0.56	0.41	0.41	1.09
Limestone, Fine	0.40	0.40	0.40	0.73
DL-Methionine 99%	0.35	0.25	0.25	0.20
Na bicarbonate	0.16	0.19	0.19	0.28
L-Lysine HCl, 78.8%	0.14	0.14	0.14	0.15
NaCl	0.22	0.23	0.23	0.22
Filler	0.35	0.35	-	-
L-Threonine, 98%	0.10	0.08	0.08	0.07
L-Valine, 98%	-	-	-	0.01
Mineral premix *	0.10	0.10	0.10	0.05
Vitamin premix **	0.05	0.05	0.05	0.05
Coccidiostat ***	0.05	0.05	0.05	0.05
Total	100	100	100	100
Calculated composition, % unless otherwise noted				
Metabolizable energy, kcal/kg	3025	3100	3100	3175
Crude protein, %	23.38	20.96	20.99	18.17
Ca, %	0.90	0.70	0.70	0.64
Na, %	0.18	0.18	0.18	0.18
Available P, %	0.45	0.35	0.35	0.32
Methionine + cystine, digestible %	0.95	0.84	0.84	0.74
Lysine, digestible %	1.28	1.14	1.14	1.00
Threonine, digestible %	0.84	0.75	0.75	0.66

* The mineral premix supplied (per kg of diet): manganese, 100 mg; zinc, 100 mg; copper, 15 mg; iron, 15 mg; iodide; 1.2 mg; selenium, 0.25 mg. ** The vitamin premix contained (per kg of diet): vitamin A, 16,538 IU; vitamin D3, 7166 IU; vitamin E, 110 IU; vitamin B12 0.03 mg; menadione, 3.00 mg; riboflavin, 13.23 mg; d-pantothenic acid, 19.85 mg; niacin, 88.20 mg; folic acid, 1.76 mg; pyridoxine, 5.51 mg; thiamine, 3.09 mg. *** Provided 66 g of salinomycin per ton of feed for the control of coccidiosis.

**Table 3:** Categories for clinical lameness, culling, and mortality evaluation in the study.

Category	Description
Clinically Lame *	Euthanasia/culling from clinical lameness diagnosis
Culls	Euthanasia/cull from health/welfare issues, or reflecting additional sampling protocols unrelated to the study
Mortality	Mortality/death from undeterminable cause and/or presumptive sudden death syndrome

Note: * Rarely, cases of “Kinky Back” (Spondylitis/Spondylolisthesis—immobile birds in hock-sitting posture) can be observed and are considered clinical lameness in diagnosis, followed by confirmation of tibial/femoral BCO lesion(s).

**Table 4:** Progression of cumulative lameness incidence (in %) from d28 to d56 of the study.

Day	T1 ^+^	T2	T3	T4
28	0.0	0.0	0.0	0.0
29	0.0	0.0	0.0	0.0
30	1.0	0.0	0.0	0.5
31	4.0	0.5	0.5	1.5
32	4.0	1.5	0.5	2.5
33	6.0	2.5	2.0	2.5
34	7.0	6.0	2.0	4.5
35	7.0	6.0	2.0	4.5
36	12.0	10.5	2.5	6.0
37	17.0	12.0	7.0	7.5
38	23.0	17.5	10.5	9.0
39	23.0	17.5	10.5	9.0
40	30.0	22.0	13.5	11.5
41	30.0	22.0	13.5	11.5
42	30.0	22.0	13.5	11.5
43	40.0	27.0	19.5	15.5
44	48.0	34.0	26.0	18.0
45	54.0	44.5	33.5	23.5
46	54.0	44.5	33.5	23.5
47	60.0	48.5	37.0	25.5
48	66.0	56.0	45.0	28.5
49	72.0	64.0	53.5	32.0
50	72.0	64.0	53.5	32.0
51	72.0	64.0	53.5	32.0
52	78.0	70.0	63.0	38.5
53	78.0	70.0	63.0	39.0
54	83.0	78.5	73.5	46.0
55	87.0	81.0	82.5	51.5
56	88.0 ^a+^	85.0 ^a^	85.5 ^a^	55.5 ^b^

Note: Non-connecting superscripts denote significant difference. ^+^ T1 statistics provided for contextual purposes only. Treatments: T1—Positive Control (basal diet); T2—Negative Control (basal diet); T3—Basal + 1 µg/kg feed 1,25(OH)_2_D_3_-glycosides d1–d14; T4—Basal + 1 µg/kg feed 1,25(OH)_2_D_3_-glycosides d1–d28.

**Table 5:** Total clinically lame, mortality, and healthy birds at the end of the experiment on d56.

Treatment	Pen	Clinically Lame (Lame = Yes)	Mortalities/Culls (Lame = No)	Clinically Healthy (Lame = No)	Total
T1 ^+^	W1	45	5	0	50
	W2	43	5	2	50
	Total	88	10	2	100
T2	1	39	2	9	50
	12	46	3	1	50
	15	42	2	6	50
	18	43	3	4	50
	Total	170	10	20	200
T3	2	42	2	6	50
	7	43	2	5	50
	22	45	2	3	50
	24	41	3	6	50
	Total	171	9	20	200
T4	4	26	3	21	50
	8	28	2	20	50
	14	26	2	22	50
	17	31	2	17	50
	Total	111	9	80	200

Note: ^+^ T1 statistics provided for contextual purposes only. Treatments: T1—Positive Control (basal diet); T2—Negative Control (basal diet); T3—Basal + 1 µg/kg feed 1,25(OH)_2_D_3_-glycosides d1–d14; T4—Basal + 1 µg/kg feed 1,25(OH)_2_D_3_-glycosides d1–d28.

**Table 6:** Experimental broiler mortality categories (in %) for all treatments in the study (d1–d56).

Category	T1 ^+^	T2	T3	T4
Clinically Lame	88.0 ^a+^	85.0 ^a^	85.5 ^a^	55.5 ^b^
Mortalities/Culls	10.0	5.0	4.5	4.5
Clinically Healthy (Survivors)	2.0	10.0	10.0	40.0

Note: Non-connecting superscripts denote significant difference. ^+^ T1 statistics provided for contextual purposes only. Treatments: T1—Positive Control (basal diet); T2—Negative Control (basal diet); T3—Basal + 1 µg/kg feed 1,25(OH)_2_D_3_-glycosides d1–d14; T4—Basal + 1 µg/kg feed 1,25(OH)_2_D_3_-glycosides d1–d28.

**Table 7:** Binomial logistic regression of cumulative lameness incidence between experimental treatments with T2 (NC) as the reference level.

**Fixed Effect**	**Estimate**	**SE**	**95% CI**	**Odds Ratio**	***p***
Treatment					
Intercept:T2	1.73	0.20	-	-	<2.00 × 10^−16^ *
T3	0.04	0.28	−0.51, 0.59	1.0	0.89
T4	−1.5	0.24	−2.0, −1.0	0.22	5.38 × 10^−10^ *
**Random Effect**	**Variance**	**SD**			
Intercept:Pen	1.42 × 10^−9^	3.78 × 10^−5^			
**Marginal Contrast**	**Difference**	**SE**	**95% CI**		***p***
T3:T4	−0.30	0.04	−0.38, −0.22		<0.001 *

Note: Asterisk (*) denotes statistical significance. Treatments: T2—Negative Control (basal diet); T3—Basal + 1 µg/kg feed 1,25(OH)_2_D_3_-glycosides d1–d14; T4—Basal + 1 µg/kg feed 1,25(OH)_2_D_3_-glycosides d1–d28.

**Table 8:** Raw cumulative counts of BCO lesions detected in femora and tibiae of birds diagnosed as clinically lame in the study.

**Treatment**	**Right Femur**				**Left Femur**			
	**N**	**FHS**	**FHT**	**FHN**	**N**	**FHS**	**FHT**	**FHN**
T1 ^+^	46	36	5	1	42	38	5	3
T2	64	90	13	3	77	81	11	1
T3	52	100	18	1	54	99	18	0
T4	38	65	7	1	32	64	12	3
**Treatment**	**Right Tibia**				**Left Tibia**			
	**N**	**THN**		**THNS**	**N**	**THN**		**THNS**
T1 ^+^	1	38		49	1	47		40
T2	0	60		109	0	86		83
T3	0	64		107	1	95		75
T4	0	49		62	1	54		56

Note: ^+^ T1 provided for contextual purposes only. THNC excluded due to lack of observed cases. Tibial dyschondroplasia (TD) excluded (not BCO lesions). Treatments: T1—Positive Control (basal diet); T2—Negative Control (basal diet); T3—Basal + 1 µg/kg feed 1,25(OH)_2_D_3_-glycosides d1–d14; T4—Basal + 1 µg/kg feed 1,25(OH)_2_D_3_-glycosides d1–d28.

## Data Availability

All relevant data is contained within the article. The original contributions presented in the study are included in the article/Supplementary Material; further inquiries can be directed to the corresponding authors.

## Supplementary Materials

The following supporting information can be downloaded at: https://www.mdpi.com/article/10.3390/ani16162586/s1, Table S1: Censored lameness data for survival analysis of T2, T3, and T4 broilers from d21–d56; Table S2: One-way ANOVA of mean surviving broiler weights by treatment on d56.

## Abbreviations

The following abbreviations are used in this manuscript:

BCO	Bacterial Chondronecrosis with Osteomyelitis
