# Supplementary material for: Further Evaluation of 1,25(OH)2D3-Glycosides Inclusion Timing for Reducing Clinical Bacterial Chondronecrosis with Osteomyelitis-Induced Lameness Incidence in Broilers
Source: Animals (Basel). 2026 Aug 19;16(16):2586. doi: 10.3390/ani16162586 (PMC13508882; doi:10.3390/ani16162586)
Supplement: Supplementary file 1 [file animals-16-02586-s001.zip › animals-4443471-supplementary.pdf]

Table S1. Censored lameness data for survival analysis of T2, T3, and T4 broilers from d21-d56

ID	Start	End	Pen	Treatment	Lame	Diagnosis	Censor
1	21	27	24	3	N	MORTALITY	0
2	21	29	1	2	N	SAMPLING	0
3	21	29	12	2	N	SAMPLING	0
4	21	29	15	2	N	SAMPLING	0
5	21	29	18	2	N	SAMPLING	0
6	21	29	4	4	N	SAMPLING	0
7	21	29	8	4	N	SAMPLING	0
8	21	29	14	4	N	SAMPLING	0
9	21	29	17	4	N	SAMPLING	0
10	21	29	2	3	N	SAMPLING	0
11	21	29	7	3	N	SAMPLING	0
12	21	29	24	3	N	SAMPLING	0
13	21	29	22	3	N	SAMPLING	0
14	21	30	8	4	Y	LAME	1
15	21	30	4	4	N	CULL	0
16	21	31	12	2	Y	LAME	1
17	21	31	7	3	Y	LAME	1
18	21	31	17	4	Y	LAME	1
19	21	31	4	4	Y	LAME	1
20	21	31	18	2	N	CULL	0
21	21	32	18	2	Y	LAME	1
22	21	32	8	4	Y	LAME	1
23	21	32	15	2	Y	LAME	1
24	21	32	4	4	Y	LAME	1
25	21	33	12	2	Y	LAME	1
26	21	33	18	2	Y	LAME	1
27	21	33	7	3	Y	LAME	1
28	21	33	2	3	Y	LAME	1
29	21	33	24	3	Y	LAME	1
30	21	33	1	2	N	SAMPLING	0
31	21	33	12	2	N	SAMPLING	0
32	21	33	15	2	N	SAMPLING	0
33	21	33	18	2	N	SAMPLING	0
34	21	33	2	3	N	SAMPLING	0
35	21	33	7	3	N	SAMPLING	0
36	21	33	22	3	N	SAMPLING	0
37	21	33	4	4	N	SAMPLING	0
38	21	33	8	4	N	SAMPLING	0
39	21	33	14	4	N	SAMPLING	0
40	21	33	17	4	N	SAMPLING	0
41	21	33	24	3	N	SAMPLING	0
42	21	34	4	4	Y	LAME	1

43	21	34	18	2	Y	LAME	1
44	21	34	18	2	Y	LAME	1
45	21	34	15	2	Y	LAME	1
46	21	34	1	2	Y	LAME	1
47	21	34	8	4	Y	LAME	1
48	21	34	17	4	Y	LAME	1
49	21	34	1	2	Y	LAME	1
50	21	34	1	2	Y	LAME	1
51	21	34	1	2	Y	LAME	1
52	21	34	4	4	Y	LAME	1
53	21	36	18	2	Y	LAME	1
54	21	36	18	2	Y	LAME	1
55	21	36	12	2	Y	LAME	1
56	21	36	12	2	Y	LAME	1
57	21	36	1	2	Y	LAME	1
58	21	36	1	2	Y	LAME	1
59	21	36	1	2	Y	LAME	1
60	21	36	7	3	Y	LAME	1
61	21	36	15	2	Y	LAME	1
62	21	36	4	4	Y	LAME	1
63	21	36	14	4	Y	LAME	1
64	21	36	15	2	Y	LAME	1
65	21	36	8	4	Y	LAME	1
66	21	37	12	2	Y	LAME	1
67	21	37	17	4	Y	LAME	1
68	21	37	22	3	Y	LAME	1
69	21	37	22	3	Y	LAME	1
70	21	37	24	3	Y	LAME	1
71	21	37	24	3	Y	LAME	1
72	21	37	18	2	Y	LAME	1
73	21	37	7	3	Y	LAME	1
74	21	37	2	3	Y	LAME	1
75	21	37	2	3	Y	LAME	1
76	21	37	8	4	Y	LAME	1
77	21	37	7	3	Y	LAME	1
78	21	37	1	2	Y	LAME	1
79	21	37	22	3	Y	LAME	1
80	21	37	4	4	Y	LAME	1
81	21	38	22	3	Y	LAME	1
82	21	38	17	4	Y	LAME	1
83	21	38	15	2	Y	LAME	1
84	21	38	18	2	Y	LAME	1
85	21	38	18	2	Y	LAME	1
86	21	38	1	2	Y	LAME	1
87	21	38	1	2	Y	LAME	1

88	21	38	8	4	Y	LAME	1
89	21	38	24	3	Y	LAME	1
90	21	38	12	2	Y	LAME	1
91	21	38	24	3	Y	LAME	1
92	21	38	7	3	Y	LAME	1
93	21	38	12	2	Y	LAME	1
94	21	38	4	4	Y	LAME	1
95	21	38	15	2	Y	LAME	1
96	21	38	15	2	Y	LAME	1
97	21	38	2	3	Y	LAME	1
98	21	38	1	2	Y	LAME	1
99	21	38	2	3	Y	LAME	1
100	21	38	12	2	Y	LAME	1
101	21	38	7	3	Y	LAME	1
102	21	40	1	2	Y	LAME	1
103	21	40	1	2	Y	LAME	1
104	21	40	24	3	Y	LAME	1
105	21	40	12	2	Y	LAME	1
106	21	40	12	2	N	MORTALITY	0
107	21	40	12	2	Y	LAME	1
108	21	40	17	4	Y	LAME	1
109	21	40	17	4	Y	LAME	1
110	21	40	22	3	Y	LAME	1
111	21	40	22	3	Y	LAME	1
112	21	40	18	2	Y	LAME	1
113	21	40	18	2	Y	LAME	1
114	21	40	18	2	Y	LAME	1
115	21	40	15	2	Y	LAME	1
116	21	40	2	3	Y	LAME	1
117	21	40	2	3	Y	LAME	1
118	21	40	8	4	Y	LAME	1
119	21	40	14	4	Y	LAME	1
120	21	40	14	4	Y	LAME	1
121	21	40	7	3	Y	LAME	1
122	21	40	15	2	Y	LAME	1
123	21	43	18	2	Y	LAME	1
124	21	43	15	2	Y	LAME	1
125	21	43	15	2	Y	LAME	1
126	21	43	24	3	Y	LAME	1
127	21	43	24	3	Y	LAME	1
128	21	43	1	2	Y	LAME	1
129	21	43	24	3	Y	LAME	1
130	21	43	15	2	Y	LAME	1
131	21	43	18	2	Y	LAME	1
132	21	43	18	2	Y	LAME	1

133	21	43	4	4	Y	LAME	1
134	21	43	17	4	Y	LAME	1
135	21	43	2	3	Y	LAME	1
136	21	43	7	3	Y	LAME	1
137	21	43	8	4	Y	LAME	1
138	21	43	2	3	Y	LAME	1
139	21	43	2	3	Y	LAME	1
140	21	43	12	2	Y	LAME	1
141	21	43	22	3	Y	LAME	1
142	21	43	7	3	Y	LAME	1
143	21	43	7	3	Y	LAME	1
144	21	43	22	3	Y	LAME	1
145	21	43	12	2	Y	LAME	1
146	21	43	22	3	Y	LAME	1
147	21	43	12	2	Y	LAME	1
148	21	43	17	4	Y	LAME	1
149	21	43	8	4	Y	LAME	1
150	21	43	17	4	Y	LAME	1
151	21	43	4	4	Y	LAME	1
152	21	43	4	4	Y	LAME	1
153	21	44	15	2	Y	LAME	1
154	21	44	14	4	Y	LAME	1
155	21	44	24	3	Y	LAME	1
156	21	44	15	2	Y	LAME	1
157	21	44	15	2	Y	LAME	1
158	21	44	12	2	Y	LAME	1
159	21	44	1	2	Y	LAME	1
160	21	44	2	3	Y	LAME	1
161	21	44	2	3	Y	LAME	1
162	21	44	1	2	Y	LAME	1
163	21	44	1	2	Y	LAME	1
164	21	44	12	2	Y	LAME	1
165	21	44	12	2	Y	LAME	1
166	21	44	7	3	Y	LAME	1
167	21	44	12	2	Y	LAME	1
168	21	44	24	3	Y	LAME	1
169	21	44	2	3	Y	LAME	1
170	21	44	24	3	Y	LAME	1
171	21	44	14	4	Y	LAME	1
172	21	44	7	3	Y	LAME	1
173	21	44	22	3	Y	LAME	1
174	21	44	17	4	Y	LAME	1
175	21	44	18	2	Y	LAME	1
176	21	44	18	2	Y	LAME	1
177	21	44	8	4	Y	LAME	1

178	21	44	18	2	Y	LAME	1
179	21	44	22	3	Y	LAME	1
180	21	44	22	3	Y	LAME	1
181	21	44	7	3	Y	LAME	1
182	21	44	18	2	Y	LAME	1
183	21	44	7	3	Y	LAME	1
184	21	44	4	4	Y	LAME	1
185	21	45	22	3	Y	LAME	1
186	21	45	15	2	Y	LAME	1
187	21	45	7	3	Y	LAME	1
188	21	45	18	2	Y	LAME	1
189	21	45	22	3	Y	LAME	1
190	21	45	22	3	Y	LAME	1
191	21	45	15	2	Y	LAME	1
192	21	45	24	3	Y	LAME	1
193	21	45	22	3	Y	LAME	1
194	21	45	12	2	Y	LAME	1
195	21	45	18	2	Y	LAME	1
196	21	45	24	3	Y	LAME	1
197	21	45	12	2	Y	LAME	1
198	21	45	17	4	Y	LAME	1
199	21	45	17	4	Y	LAME	1
200	21	45	18	2	Y	LAME	1
201	21	45	12	2	Y	LAME	1
202	21	45	18	2	Y	LAME	1
203	21	45	2	3	Y	LAME	1
204	21	45	12	2	Y	LAME	1
205	21	45	2	3	Y	LAME	1
206	21	45	12	2	Y	LAME	1
207	21	45	17	4	Y	LAME	1
208	21	45	15	2	Y	LAME	1
209	21	45	2	3	Y	LAME	1
210	21	45	2	3	Y	LAME	1
211	21	45	1	2	Y	LAME	1
212	21	45	1	2	Y	LAME	1
213	21	45	1	2	Y	LAME	1
214	21	45	17	4	Y	LAME	1
215	21	45	24	3	Y	LAME	1
216	21	45	14	4	Y	LAME	1
217	21	45	14	4	Y	LAME	1
218	21	45	8	4	Y	LAME	1
219	21	45	4	4	Y	LAME	1
220	21	45	4	4	Y	LAME	1
221	21	47	17	4	Y	LAME	1
222	21	47	7	3	Y	LAME	1

223	21	47	18	2	Y	LAME	1
224	21	47	18	2	Y	LAME	1
225	21	47	1	2	Y	LAME	1
226	21	47	7	3	Y	LAME	1
227	21	47	2	3	Y	LAME	1
228	21	47	15	2	Y	LAME	1
229	21	47	2	3	Y	LAME	1
230	21	47	18	2	Y	LAME	1
231	21	47	1	2	Y	LAME	1
232	21	47	15	2	Y	LAME	1
233	21	47	1	2	Y	LAME	1
234	21	47	2	3	Y	LAME	1
235	21	47	14	4	Y	LAME	1
236	21	47	22	3	Y	LAME	1
237	21	47	14	4	Y	LAME	1
238	21	47	22	3	Y	LAME	1
239	21	47	8	4	Y	LAME	1
240	21	45	15	2	Y	LAME	1
241	21	45	22	3	Y	LAME	1
242	21	45	4	4	Y	LAME	1
243	21	45	17	4	Y	LAME	1
244	21	45	12	2	Y	LAME	1
245	21	45	12	2	Y	LAME	1
246	21	45	12	2	Y	LAME	1
247	21	45	12	2	Y	LAME	1
248	21	45	12	2	Y	LAME	1
249	21	45	24	3	Y	LAME	1
250	21	45	24	3	Y	LAME	1
251	21	48	1	2	Y	LAME	1
252	21	48	1	2	Y	LAME	1
253	21	48	1	2	Y	LAME	1
254	21	48	18	2	Y	LAME	1
255	21	48	15	2	Y	LAME	1
256	21	48	18	2	Y	LAME	1
257	21	48	15	2	Y	LAME	1
258	21	48	17	4	Y	LAME	1
259	21	48	17	4	Y	LAME	1
260	21	48	15	2	Y	LAME	1
261	21	48	7	3	Y	LAME	1
262	21	48	22	3	Y	LAME	1
263	21	48	12	2	Y	LAME	1
264	21	48	22	3	Y	LAME	1
265	21	48	22	3	Y	LAME	1
266	21	48	2	3	Y	LAME	1
267	21	48	24	3	Y	LAME	1

268	21	48	18	2	Y	LAME	1
269	21	48	7	3	Y	LAME	1
270	21	48	4	4	Y	LAME	1
271	21	48	7	3	Y	LAME	1
272	21	48	15	2	Y	LAME	1
273	21	48	18	2	Y	LAME	1
274	21	48	14	4	Y	LAME	1
275	21	48	8	4	Y	LAME	1
276	21	48	8	4	Y	LAME	1
277	21	48	2	3	Y	LAME	1
278	21	48	12	2	Y	LAME	1
279	21	48	24	3	Y	LAME	1
280	21	48	2	3	Y	LAME	1
281	21	48	12	2	Y	LAME	1
282	21	48	24	3	Y	LAME	1
283	21	48	12	2	Y	LAME	1
284	21	48	22	3	Y	LAME	1
285	21	48	24	3	Y	LAME	1
286	21	48	7	3	Y	LAME	1
287	21	48	7	3	Y	LAME	1
288	21	49	24	3	Y	LAME	1
289	21	49	22	3	Y	LAME	1
290	21	49	24	3	Y	LAME	1
291	21	49	24	3	Y	LAME	1
292	21	49	24	3	Y	LAME	1
293	21	49	24	3	Y	LAME	1
294	21	49	22	3	Y	LAME	1
295	21	49	22	3	Y	LAME	1
296	21	49	22	3	Y	LAME	1
297	21	49	22	3	Y	LAME	1
298	21	49	15	2	Y	LAME	1
299	21	49	15	2	Y	LAME	1
300	21	49	1	2	Y	LAME	1
301	21	49	18	2	Y	LAME	1
302	21	49	12	2	Y	LAME	1
303	21	49	18	2	Y	LAME	1
304	21	49	15	2	Y	LAME	1
305	21	49	1	2	Y	LAME	1
306	21	49	12	2	Y	LAME	1
307	21	49	12	2	Y	LAME	1
308	21	49	2	3	Y	LAME	1
309	21	49	4	4	Y	LAME	1
310	21	49	4	4	Y	LAME	1
311	21	49	14	4	Y	LAME	1
312	21	49	18	2	Y	LAME	1

313	21	49	17	4	Y	LAME	1
314	21	49	12	2	Y	LAME	1
315	21	49	14	4	Y	LAME	1
316	21	49	2	3	Y	LAME	1
317	21	49	1	2	Y	LAME	1
318	21	49	17	4	Y	LAME	1
319	21	49	7	3	Y	LAME	1
320	21	49	7	3	Y	LAME	1
321	21	52	15	2	Y	LAME	1
322	21	52	15	2	Y	LAME	1
323	21	52	1	2	Y	LAME	1
324	21	52	7	3	Y	LAME	1
325	21	52	2	3	Y	LAME	1
326	21	52	7	3	Y	LAME	1
327	21	52	2	3	Y	LAME	1
328	21	52	7	3	Y	LAME	1
329	21	52	2	3	Y	LAME	1
330	21	52	7	3	Y	LAME	1
331	21	52	2	3	Y	LAME	1
332	21	52	7	3	Y	LAME	1
333	21	52	2	3	Y	LAME	1
334	21	52	22	3	Y	LAME	1
335	21	52	12	2	Y	LAME	1
336	21	52	24	3	Y	LAME	1
337	21	52	12	2	Y	LAME	1
338	21	52	12	2	Y	LAME	1
339	21	52	22	3	Y	LAME	1
340	21	52	22	3	Y	LAME	1
341	21	52	12	2	Y	LAME	1
342	21	52	22	3	Y	LAME	1
343	21	52	24	3	Y	LAME	1
344	21	52	12	2	Y	LAME	1
345	21	52	24	3	Y	LAME	1
346	21	52	24	3	Y	LAME	1
347	21	52	14	4	Y	LAME	1
348	21	52	8	4	Y	LAME	1
349	21	52	8	4	Y	LAME	1
350	21	52	8	4	Y	LAME	1
351	21	52	4	4	Y	LAME	1
352	21	52	22	3	Y	LAME	1
353	21	52	4	4	Y	LAME	1
354	21	52	14	4	Y	LAME	1
355	21	52	14	4	Y	LAME	1
356	21	52	14	4	Y	LAME	1
357	21	52	17	4	Y	LAME	1

358	21	52	17	4	Y	LAME	1
359	21	52	17	4	Y	LAME	1
360	21	53	4	4	Y	LAME	1
361	21	54	15	2	Y	LAME	1
362	21	54	15	2	Y	LAME	1
363	21	54	15	2	Y	LAME	1
364	21	54	15	2	Y	LAME	1
365	21	54	15	2	Y	LAME	1
366	21	54	2	3	Y	LAME	1
367	21	54	2	3	Y	LAME	1
368	21	54	1	2	Y	LAME	1
369	21	54	8	4	Y	LAME	1
370	21	54	18	2	Y	LAME	1
371	21	54	1	2	Y	LAME	1
372	21	54	1	2	Y	LAME	1
373	21	54	18	2	Y	LAME	1
374	21	54	2	3	Y	LAME	1
375	21	54	18	2	Y	LAME	1
376	21	54	2	3	Y	LAME	1
377	21	54	1	2	Y	LAME	1
378	21	54	18	2	Y	LAME	1
379	21	54	14	4	Y	LAME	1
380	21	54	14	4	Y	LAME	1
381	21	54	14	4	Y	LAME	1
382	21	54	14	4	Y	LAME	1
383	21	54	4	4	Y	LAME	1
384	21	54	4	4	Y	LAME	1
385	21	54	14	4	Y	LAME	1
386	21	54	17	4	Y	LAME	1
387	21	54	7	3	Y	LAME	1
388	21	54	17	4	Y	LAME	1
389	21	54	17	4	Y	LAME	1
390	21	54	8	4	Y	LAME	1
391	21	54	12	2	Y	LAME	1
392	21	54	12	2	Y	LAME	1
393	21	54	8	4	Y	LAME	1
394	21	54	24	3	Y	LAME	1
395	21	54	24	3	Y	LAME	1
396	21	54	24	3	Y	LAME	1
397	21	54	8	4	Y	LAME	1
398	21	54	24	3	Y	LAME	1
399	21	54	22	3	Y	LAME	1
400	21	54	22	3	Y	LAME	1
401	21	54	22	3	Y	LAME	1
402	21	55	14	4	Y	LAME	1

403	21	55	14	4	Y	LAME	1
404	21	55	14	4	Y	LAME	1
405	21	56	15	2	Y	LAME	1
406	21	56	17	4	Y	LAME	1
407	21	56	15	2	Y	LAME	1
408	21	56	15	2	Y	LAME	1
409	21	56	4	4	Y	LAME	1
410	21	56	17	4	Y	LAME	1
411	21	56	2	3	Y	LAME	1
412	21	56	1	2	Y	LAME	1
413	21	56	1	2	Y	LAME	1
414	21	56	2	3	Y	LAME	1
415	21	56	4	4	Y	LAME	1
416	21	56	18	2	Y	LAME	1
417	21	56	18	2	Y	LAME	1
418	21	56	12	2	Y	LAME	1
419	21	56	24	3	Y	LAME	1
420	21	56	14	4	Y	LAME	1
421	21	56	22	3	Y	LAME	1
422	21	56	8	4	Y	LAME	1
423	21	56	14	4	Y	LAME	1
424	21	56	7	3	Y	LAME	1
425	21	56	7	3	Y	LAME	1
426	21	56	8	4	Y	LAME	1
427	21	49	7	3	Y	LAME	1
428	21	49	7	3	Y	LAME	1
429	21	49	8	4	Y	LAME	1
430	21	49	18	2	Y	LAME	1
431	21	49	2	3	Y	LAME	1
432	21	49	1	2	Y	LAME	1
433	21	49	15	2	Y	LAME	1
434	21	52	1	2	Y	LAME	1
435	21	52	18	2	Y	LAME	1
436	21	52	18	2	Y	LAME	1
437	21	52	15	2	Y	LAME	1
438	21	52	8	4	Y	LAME	1
439	21	54	22	3	Y	LAME	1
440	21	54	7	3	Y	LAME	1
441	21	54	7	3	Y	LAME	1
442	21	54	7	3	Y	LAME	1
443	21	54	7	3	Y	LAME	1
444	21	54	22	3	Y	LAME	1
445	21	54	7	3	Y	LAME	1
446	21	54	12	2	Y	LAME	1
447	21	54	22	3	Y	LAME	1

448	21	54	12	2	Y	LAME	1
449	21	54	22	3	Y	LAME	1
450	21	55	15	2	Y	LAME	1
451	21	55	2	3	Y	LAME	1
452	21	55	12	2	Y	LAME	1
453	21	55	2	3	Y	LAME	1
454	21	55	2	3	Y	LAME	1
455	21	55	18	2	Y	LAME	1
456	21	55	24	3	Y	LAME	1
457	21	55	2	3	Y	LAME	1
458	21	55	22	3	Y	LAME	1
459	21	55	22	3	Y	LAME	1
460	21	55	7	3	Y	LAME	1
461	21	55	15	2	Y	LAME	1
462	21	55	2	3	Y	LAME	1
463	21	55	22	3	Y	LAME	1
464	21	55	24	3	Y	LAME	1
465	21	55	24	3	Y	LAME	1
466	21	55	1	2	Y	LAME	1
467	21	55	8	4	Y	LAME	1
468	21	55	24	3	Y	LAME	1
469	21	55	7	3	Y	LAME	1
470	21	55	24	3	Y	LAME	1
471	21	55	22	3	Y	LAME	1
472	21	55	8	4	Y	LAME	1
473	21	55	24	3	Y	LAME	1
474	21	55	17	4	Y	LAME	1
475	21	55	7	3	Y	LAME	1
476	21	55	4	4	Y	LAME	1
477	21	55	17	4	Y	LAME	1
478	21	55	4	4	Y	LAME	1
479	21	55	8	4	Y	LAME	1
480	21	55	17	4	Y	LAME	1
481	21	56	1	2	N	HEALTHY	0
482	21	56	1	2	N	HEALTHY	0
483	21	56	1	2	N	HEALTHY	0
484	21	56	1	2	N	HEALTHY	0
485	21	56	1	2	N	HEALTHY	0
486	21	56	1	2	N	HEALTHY	0
487	21	56	1	2	N	HEALTHY	0
488	21	56	1	2	N	HEALTHY	0
489	21	56	1	2	N	HEALTHY	0
490	21	56	2	3	N	HEALTHY	0
491	21	56	2	3	N	HEALTHY	0
492	21	56	2	3	N	HEALTHY	0

493	21	56	2	3	N	HEALTHY	0
494	21	56	2	3	N	HEALTHY	0
495	21	56	2	3	N	HEALTHY	0
496	21	56	4	4	N	HEALTHY	0
497	21	56	4	4	N	HEALTHY	0
498	21	56	4	4	N	HEALTHY	0
499	21	56	4	4	N	HEALTHY	0
500	21	56	4	4	N	HEALTHY	0
501	21	56	4	4	N	HEALTHY	0
502	21	56	4	4	N	HEALTHY	0
503	21	56	4	4	N	HEALTHY	0
504	21	56	4	4	N	HEALTHY	0
505	21	56	4	4	N	HEALTHY	0
506	21	56	4	4	N	HEALTHY	0
507	21	56	4	4	N	HEALTHY	0
508	21	56	4	4	N	HEALTHY	0
509	21	56	4	4	N	HEALTHY	0
510	21	56	4	4	N	HEALTHY	0
511	21	56	4	4	N	HEALTHY	0
512	21	56	4	4	N	HEALTHY	0
513	21	56	4	4	N	HEALTHY	0
514	21	56	4	4	N	HEALTHY	0
515	21	56	4	4	N	HEALTHY	0
516	21	56	4	4	N	HEALTHY	0
517	21	56	7	3	N	HEALTHY	0
518	21	56	7	3	N	HEALTHY	0
519	21	56	7	3	N	HEALTHY	0
520	21	56	7	3	N	HEALTHY	0
521	21	56	7	3	N	HEALTHY	0
522	21	56	8	4	N	HEALTHY	0
523	21	56	8	4	N	HEALTHY	0
524	21	56	8	4	N	HEALTHY	0
525	21	56	8	4	N	HEALTHY	0
526	21	56	8	4	N	HEALTHY	0
527	21	56	8	4	N	HEALTHY	0
528	21	56	8	4	N	HEALTHY	0
529	21	56	8	4	N	HEALTHY	0
530	21	56	8	4	N	HEALTHY	0
531	21	56	8	4	N	HEALTHY	0
532	21	56	8	4	N	HEALTHY	0
533	21	56	8	4	N	HEALTHY	0
534	21	56	8	4	N	HEALTHY	0
535	21	56	8	4	N	HEALTHY	0
536	21	56	8	4	N	HEALTHY	0
537	21	56	8	4	N	HEALTHY	0

538	21	56	8	4	N	HEALTHY	0
539	21	56	8	4	N	HEALTHY	0
540	21	56	8	4	N	HEALTHY	0
541	21	56	8	4	N	HEALTHY	0
542	21	56	12	2	N	HEALTHY	0
543	21	56	14	4	N	HEALTHY	0
544	21	56	14	4	N	HEALTHY	0
545	21	56	14	4	N	HEALTHY	0
546	21	56	14	4	N	HEALTHY	0
547	21	56	14	4	N	HEALTHY	0
548	21	56	14	4	N	HEALTHY	0
549	21	56	14	4	N	HEALTHY	0
550	21	56	14	4	N	HEALTHY	0
551	21	56	14	4	N	HEALTHY	0
552	21	56	14	4	N	HEALTHY	0
553	21	56	14	4	N	HEALTHY	0
554	21	56	14	4	N	HEALTHY	0
555	21	56	14	4	N	HEALTHY	0
556	21	56	14	4	N	HEALTHY	0
557	21	56	14	4	N	HEALTHY	0
558	21	56	14	4	N	HEALTHY	0
559	21	56	14	4	N	HEALTHY	0
560	21	56	14	4	N	HEALTHY	0
561	21	56	14	4	N	HEALTHY	0
562	21	56	14	4	N	HEALTHY	0
563	21	56	14	4	N	HEALTHY	0
564	21	56	14	4	N	HEALTHY	0
565	21	56	15	2	N	HEALTHY	0
566	21	56	15	2	N	HEALTHY	0
567	21	56	15	2	N	HEALTHY	0
568	21	56	15	2	N	HEALTHY	0
569	21	56	15	2	N	HEALTHY	0
570	21	56	15	2	N	HEALTHY	0
571	21	56	17	4	N	HEALTHY	0
572	21	56	17	4	N	HEALTHY	0
573	21	56	17	4	N	HEALTHY	0
574	21	56	17	4	N	HEALTHY	0
575	21	56	17	4	N	HEALTHY	0
576	21	56	17	4	N	HEALTHY	0
577	21	56	17	4	N	HEALTHY	0
578	21	56	17	4	N	HEALTHY	0
579	21	56	17	4	N	HEALTHY	0
580	21	56	17	4	N	HEALTHY	0
581	21	56	17	4	N	HEALTHY	0
582	21	56	17	4	N	HEALTHY	0

583	21	56	17	4	N	HEALTHY	0
584	21	56	17	4	N	HEALTHY	0
585	21	56	17	4	N	HEALTHY	0
586	21	56	17	4	N	HEALTHY	0
587	21	56	17	4	N	HEALTHY	0
588	21	56	18	2	N	HEALTHY	0
589	21	56	18	2	N	HEALTHY	0
590	21	56	18	2	N	HEALTHY	0
591	21	56	18	2	N	HEALTHY	0
592	21	56	22	3	N	HEALTHY	0
593	21	56	22	3	N	HEALTHY	0
594	21	56	22	3	N	HEALTHY	0
595	21	56	24	3	N	HEALTHY	0
596	21	56	24	3	N	HEALTHY	0
597	21	56	24	3	N	HEALTHY	0
598	21	56	24	3	N	HEALTHY	0
599	21	56	24	3	N	HEALTHY	0
600	21	56	24	3	N	HEALTHY	0

Note: Diagnosis categories as follows: MORTALITY = Death from natural/indeterminable causes; SAMPLING = Culled for sampling purposes; CULL = Culled for non-lame health/welfare issues; LAME = Clinical lameness diagnosis; HEALTHY = Surviving, clinically healthy birds at the end of the experiment. Censor code: 0 = Censored (events not considered clinical lameness, Lame = NO); 1 = Event (clinical lameness diagnosis, Lame = YES). Treatments: T2 – Negative Control (basal diet); T3 – Basal + 1 μg/kg feed 1,25(OH)2D3-glycosides d1–d14; T4 – Basal + 1 μg/kg feed 1,25(OH)2D3-glycosides d1–d28.

Table S2. One-way ANOVA of mean surviving broiler weights by treatment on d56

Treatment	Pens	Remaining Birds (*n*)	d56 Avg Weight (kg) ± SD
T1^{+}	2^{+}	2^{+}	4.344 ± 0.136 $^{b+}$
T2	4	20	4.857 ± 0.070 ^{a}
T3	4	20	4.753 ± 0.309 ^{ab}
T4	4	80	4.935 ± 0.097 ^{a}

Note: Non-connecting superscripts denote significant difference. Surviving birds were weighed on a pen average basis, followed by ANOVA of treatment means. $^{+}$T1 statistics (evaluated with other treatments through nonparametric Kruskal-Wallis test) provided for contextual purposes only. All remaining treatments were evaluated with ANOVA and reported no significant difference. Treatments: T1 – Positive Control (basal diet); T2 – Negative Control (basal diet); T3 – Basal + 1 μg/kg feed 1,25$(OH)_2D_3$-glycosides d1–d14; T4 – Basal + 1 μg/kg feed 1,25$(OH)_2D_3$-glycosides d1–d28
